# Close social relationships and happiness in the United States: the moderating role of love by God

**DOI:** 10.3389/fpubh.2025.1567701

**Published:** 2025-05-14

**Authors:** Stephanie Moller

**Affiliations:** Department of Sociology, University of North Carolina at Charlotte, Charlotte, NC, United States

**Keywords:** love, happiness, close relationship, God, happiness and wellbeing

## Abstract

In the contemporary United States, many individuals suffer from a lack of close relationships, negatively affecting their happiness. At the same time, many individuals do not feel a loving relationship with God/Spirit (i.e., religious love). In the 1950s, theorist Pitirim Sorokin posited that love, particularly religious love, acts as a transformative energy capable of fostering resilience in the modern rational world, and scholars hypothesized a link between religious love and happiness. However, the topic remains understudied. This study analyzes United States data from the Global Flourishing Study and presents results from linear regression with robust standard errors. The findings indicate that a central component of religious love, love by God, mitigates the adverse effects of lacking close relationships. This study calls on researchers to further investigate the significant yet understudied role of religious love in individuals’ lives.

## 1 Introduction

Sociologists and social scientists have long recognized the importance of emotions in shaping individual interactions and societal structures (63, 64). However, the empirical examination of love within society remains limited, with most studies focusing on romantic, parental, or sexual love (1). Research on religious love—love by God or the Divine—is particularly scarce. Yet, Sorokin’s (2) theory of love suggests that religious love has the potential to foster resilience to societal stressors and ultimately promote greater happiness.

One notable social stressor is the absence of close relationships. Extensive research highlights the critical role of close social relationships in fostering happiness (3–5). These relationships act as buffers during stressful situations (6, 7). Most studies on close relationships explore their supportive versus detrimental characteristics, their evolution across the life course, and their impact on wellbeing, happiness, and health (7–9).

Despite these advancements, scholars have rarely assessed whether the relationship between close relationships and happiness is influenced by perceptions of love by God. This study proposes that love by God may serve as a buffer, protecting individuals without close relationships from the negative effects on happiness. Using U.S. data from the Global Flourishing Study, this research examines whether love by God moderates the relationship between close social relationships and happiness. This inquiry is especially relevant in light of two pressing trends in the United States: (1) increased social disconnectedness, and (2) declining belief in God (10, 11). The tested hypothesis was preregistered with OSF Registries at: https://osf.io/rz23g.

### 1.1 Relationships, love by God, and happiness

Pitirim Sorokin developed a multidimensional theory of love, which included religious love, conceptualized as love by God or a divine, spiritual being (12, 13). While religions have created distinct traditions to honor this form of love, Sorokin argued that religious love itself is universal. He described it as a transformative energy, capable of reshaping humanity by fostering love, compassion, and a sense of unity with both the divine and others (14, 15). Sorokin further theorized that modernization has shifted society away from “ideational truth,” which incorporates supraconscious, spiritual perceptions, toward “sensate truth,” which relies on sensory perception and measurable reality (16). This transition, he contended, has led to a decline in social harmony and integration, as humanity lost its connection to supraconscious love (17). Similarly, scholars argue that capitalism and modern development are antithetical to love, as the focus on reason and efficiency undermines human relationships (18). According to Sorokin, love in general, and religious love in particular, serves as a force for social integration (19), as God is a member of a person’s social network that can offer support and even combat loneliness (20).

The lack of harmony and social integration, as theorized by Sorokin, becomes particularly evident when individuals lack close, supportive interpersonal relationships. In fact, love is often defined as a desire to have a close connected ongoing relationship with another (21). Thus, close relationships are built on a foundation of love, even when the actors do not consciously acknowledge the underlying love. Indeed, Sternberg (22) identifies three components of love: intimacy, commitment, and passion, two of which are stable in most close relationships. The first component, intimacy, represents connectedness and bondedness. It is reflected in the desire to support another and the ability to count on another. The second component of love, commitment, reflects the decision to be committed to another, though the length of the commitment is variable. Thus, close social relationships are founded upon love. This conception of love aligns with Sorokin’s broader conceptualization of love which includes psychological love—empathy, devotion, and respect–and social love—including interconnectedness and caring for others (23, 24).

Close relationships are associated with greater wellbeing, happiness, and health outcomes (5, 25–33). Researchers have emphasized that social connection is essential for humans, as it motivates behavior and enhances wellbeing (34). Importantly, some scholars suggest that the mere existence of at least one meaningful social bond is more critical to happiness than the number of relationships (4). Conversely, a lack of close relationships is a key predictor of poor health, diminished wellbeing, and unhappiness (35–38).

Despite these findings, no research has examined whether religious love, specifically love by God, moderates the association between close social relationships and happiness. Sorokin theorized that religious love fosters social integration and cohesion, which in turn enhances happiness. This idea is supported by studies showing that religious love promotes health and wellbeing, including happiness, and protects against poor health and psychological distress (1, 39, 40).

Religious love may moderate the association between personal relationships and happiness because, as Sorokin posited, it serves as a transformative energy. In this sense, God or Spirit acts as an omnipresent force, guiding individuals’ interactions, perceptions, and behaviors across social contexts. Dill (41), for instance, found in a qualitative study of Black youth that their faith and relationship with God provided a sense of protection, allowing them to turn their worries over to God. Similarly, Upenieks et al. (42) observed in a study of student-athletes that a positive relationship with God helped mitigate the negative impact of low trait courage on wellbeing. While this study did not explicitly examine love, it underscores how a relationship with God can foster resilience.

Building on these findings, this study proposes that individuals who lack close, supportive social relationships may not necessarily experience unhappiness if they perceive love by God. When traditional social support is absent, the perception of being loved by God or Spirit may offer a sense of protection and connection. Religious love, therefore, has the potential to provide resilience against the adverse effects of social disconnection. Therefore, the study assesses the following hypothesis:

Love by God will moderate the relationship between close relationships and happiness.

## 2 Methods

To assess the hypothesis, this study presents results from analysis of United States data from the first wave of the Global Flourishing Study, fielded in 2023 by Gallup, Inc. (43, 44). The Global Flourishing study was developed through a collaboration of researchers at Harvard University, Baylor University, Gallup, Inc., and the Center for Open Science. The United States data were collected with a probability-based sample of the civilian non-institutionalized population of adults, utilizing the Gallup web-based panel. Gallup, Inc. recruited panelists based on address or random digit dialing, with consideration of population characteristics based on census data (45). All members of the Gallup Panel were sent up to five invitations to participate in the survey. The United States response rate was 100%. The survey was administered in English. Gallup ensured quality responses by flagging surveys with: illogical or inconsistent responses, single response categories across several questions, and short time to completion. Surveys that presented multiple quality issues were excluded. Gallup also had security protocols in place to ensure that responses were unique and from valid devices. Sample weights were employed to adjust for the probability of selection and non-response (45).

The analyses focused on three primary variables. The dependent variable, happiness, was measured with the question, “In general, how happy or unhappy do you usually feel? “Responses were coded on an 11 point scale, where 0 represents extremely unhappy and 10 represents extremely happy. The primary independent variables were:

1. *Close relationships:* respondents were asked, “Is there any one special person you know that you feel very close to? For example, someone you can confide in and share your feelings with.” Responses were coded as 1 for No (no close relationship) and 0 for Yes.
2. *Loved by God*: respondents were asked, “I feel loved or cared for by God, the main God I worship, or the spiritual force that guides my life.” Loved by God was coded as 1 for Agree and 0 for Disagree, Unsure, or Not Relevant.

All models included controls for a series of variables related to both happiness and wellbeing which is a closely related construct (see Appendix A for the full list of variables). The literature has established that the quality of parental relationships predicts overall wellbeing (46–48). The quality of parental relationships was measured with two variables: relationship with mother and relationship with father. Both variables were measured on a 5-point scale, ranging from very bad to very good and were included in the analyses as categorical variables. For both variables, there was a category for “does not apply” to account for absent parents. The models also included a control for a measure of *social support*, where respondents reported the extent that they could count on people to help if they were in trouble, coded on an 11-point scale, ranging from 0 to 10.

The models also controlled for sense of country belonging, ranging from weak (0) to very strong (10) on an 11-point scale. At the country-level, sense of belonging is important for residents, particularly migrants undergoing an acculturation process (49, 50). A lack of country-connectedness can generate lower wellbeing (51). The models also controlled for *perceived discrimination* (coded as never, rarely, often, always, and missing) because perceptions of discrimination may impede social connections and wellbeing, particularly for individuals in marginalized positions (52).

Extensive research has found a relationship between religiosity/spirituality and both happiness and wellbeing (53–59). To ensure that the measure of loved by God was independent of overall religiosity, the study included controls for three measures: *Religious service attendance,* measured on a five-point scale ranging from never (1; the excluded category) to more than once a week (5); *Frequency of prayer or meditation,* measured on a four-point scale ranging from never (1, the excluded category) to more than once a day (4); and *Religiosity,* an index of four variables. The Global Flourishing Study incorporated measures that capture religiosity from a variety of previously developed and extensively tested scales (43). The religiosity measure included in this study was created with four variables. The first measure, religious beliefs and practices lie behind approach to life, was originally measured with four categories: agree, disagree, not relevant or unsure. It was recoded into a dichotomous measure of agree (1) versus disagree, not relevant, or unsure (0). The second measure, religion is an important part of daily life, was originally measured as yes, no, do not know, and refused. The variable was recoded to yes (1) versus no or do not know (0). Refused was recoded to missing. The third measure, finds strength or comfort in religion or spirituality, was originally measured as agree, disagree, not relevant, or unsure. This measure was dichotomized to compare agree (1) to disagree, not relevant, and unsure (0). The final measure, feels connected to a religion or form of spirituality was measured on a four-point scale, ranging from never (0) to always (4). A categorical principal component analysis demonstrated that the items fit together on a single construct with an eigenvalue above 1.0 and an alpha of 0.89. A factor analysis on a polychoric correlation matrix showed that the variables fit on a single factor with factor loadings for all variables above 0.9.

This study also included a control for parental love, one of the most studied forms of love. This variable was measured with three categories: Not Loved (excluded category), Loved by One Parent, and Loved by Two Parents. Parental love was created from two questions, (1) in general, did you feel loved by your mother when you were growing up? And (2) in general, did you feel loved by your father when you were growing up?

The final set of control variables included characteristics of individuals and families. Controls included age, gender (male, female, and other), foreign-born, race/ethnicity (White Non-Hispanic, Black Non-Hispanic, Asian Non-Hispanic, other and mixed race Non-Hispanic, and Hispanic) employment status (employed for an employer, self-employed, retired, unemployed, and out of the labor force), highest education (no high school diploma, high school graduate, Associate’s degree, Bachelor’s degree, and advanced degree), place (rural, small town, large city, and suburb), marital status (single and never married, married, separated or divorced, widowed, and domestic partner), and household income (categorized). See Appendix A for details and descriptive statistics.

The original data included responses from 38,312 individuals residing in the United States. The final working sample included 37,641 participants, after excluding 671 participants who were missing values on the dependent variable, the two primary independent variables or any continuous control variable. For all categorical control variables, an extra category for missing data was included. Descriptive statistics for all variables are included in Appendix A. Data were weighted with sample weights and are analyzed via linear regression with robust standard errors via the svy: regress command in STATA.

## 3 Results

Model 1 in Table 1 presents the results from a linear regression with robust standard errors, showing the direct effects of no close relationship and loved by God on happiness. The analysis shows a negative association between lacking close relationships and happiness, highlighting lower happiness levels among those without close personal connections. Conversely, individuals who perceived Love by God reported higher levels of happiness. Model 2 presents analysis that examines whether loved by God moderates the relationship between close relationships and happiness. The results show a positive and significant moderation effect, supporting the hypothesis that perceived love by God moderates the relationship between close personal relationships and happiness.

**Table 1:** Slopes and robust standard errors from linear regressions predicting happiness.

Variables	Model 1	Model 2
No close relationship	−0.434^***^ (0.07)	−0.553^***^ (0.10)
Loved by God	0.116^†^ (0.07)	0.077 (0.07)
No close relationship * loved by God	–	0.269^*^ (0.14)

^†^*p* < 0.05 (one-tailed test), *p < 0.05 (two-tailed test), **p < 0.01 (two-tailed test), ***p < 0.001 (two-tailed test); Weighted; *n* = 37,641; Controls for all variables in Appendix A.

**Figure 1:**
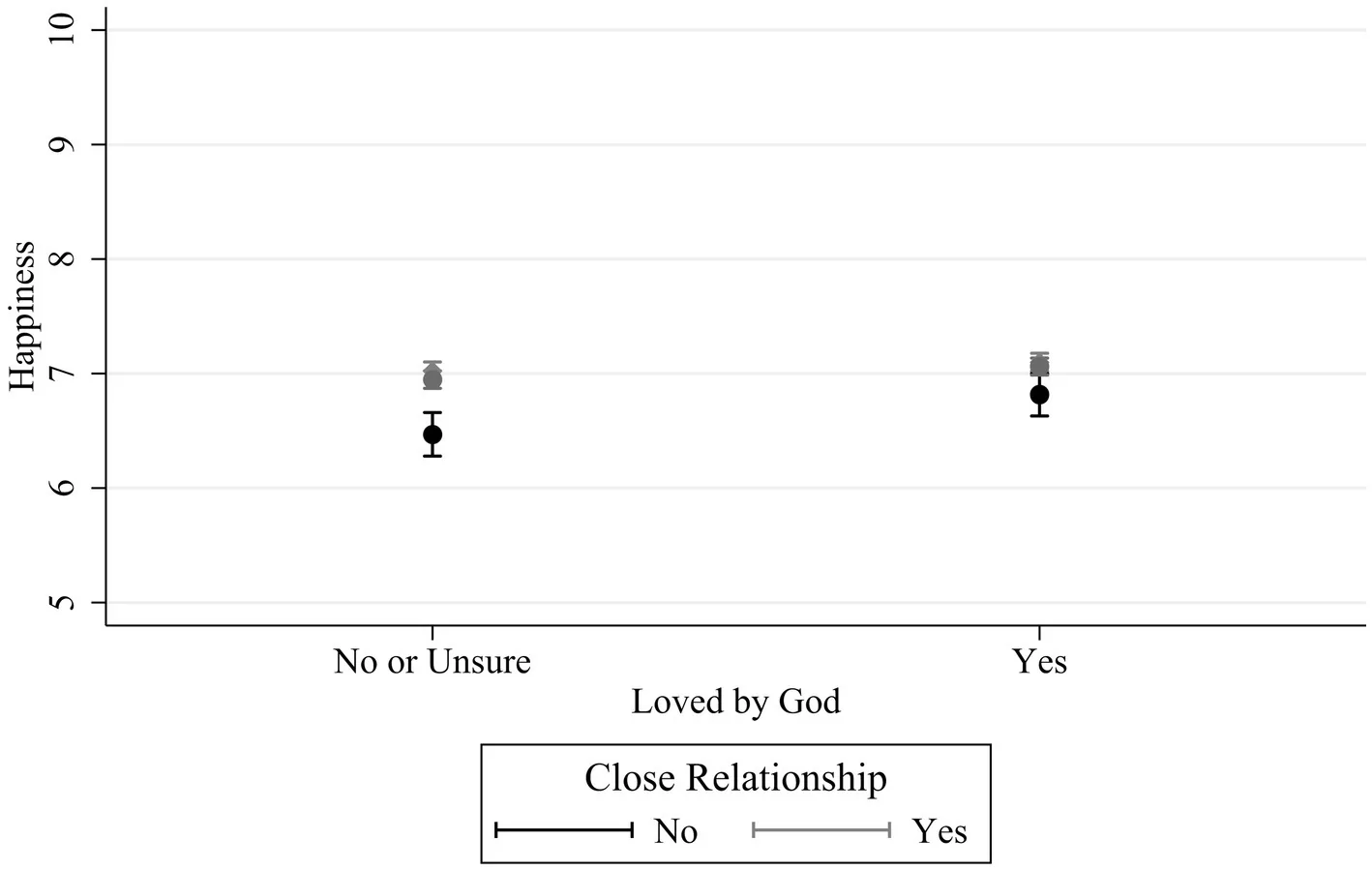
Predicted happiness by love by God and close relationship. With 95% confidence intervals; weighted.

Figure 1 illustrates predicted happiness based on perceptions of Love by God and the presence of a close relationship, while controlling for all other variables at their mean values. The x-axis categorizes individuals by whether they perceived Love by God, and the y-axis represents predicted happiness scores derived from the analysis in Table 1, Model 2. The grouping variable represents the presence or absence of a close relationship.

This graph illustrates how Love by God moderates the relationship between close social relationships and happiness. When individuals perceived love by God, there is not a significant difference in the effect that a close social relationship has on happiness. Yet, when the individual did not perceive Love by God, individuals who lacked a close social relationship had lower predicted happiness. These findings highlight the protective role of perceiving love by God, particularly for individuals who lacked close social relationships. This moderation effect demonstrates how Love by God can compensate for the absence of close social relations, fostering resilience and generating higher levels of happiness.

**Figure 2:**
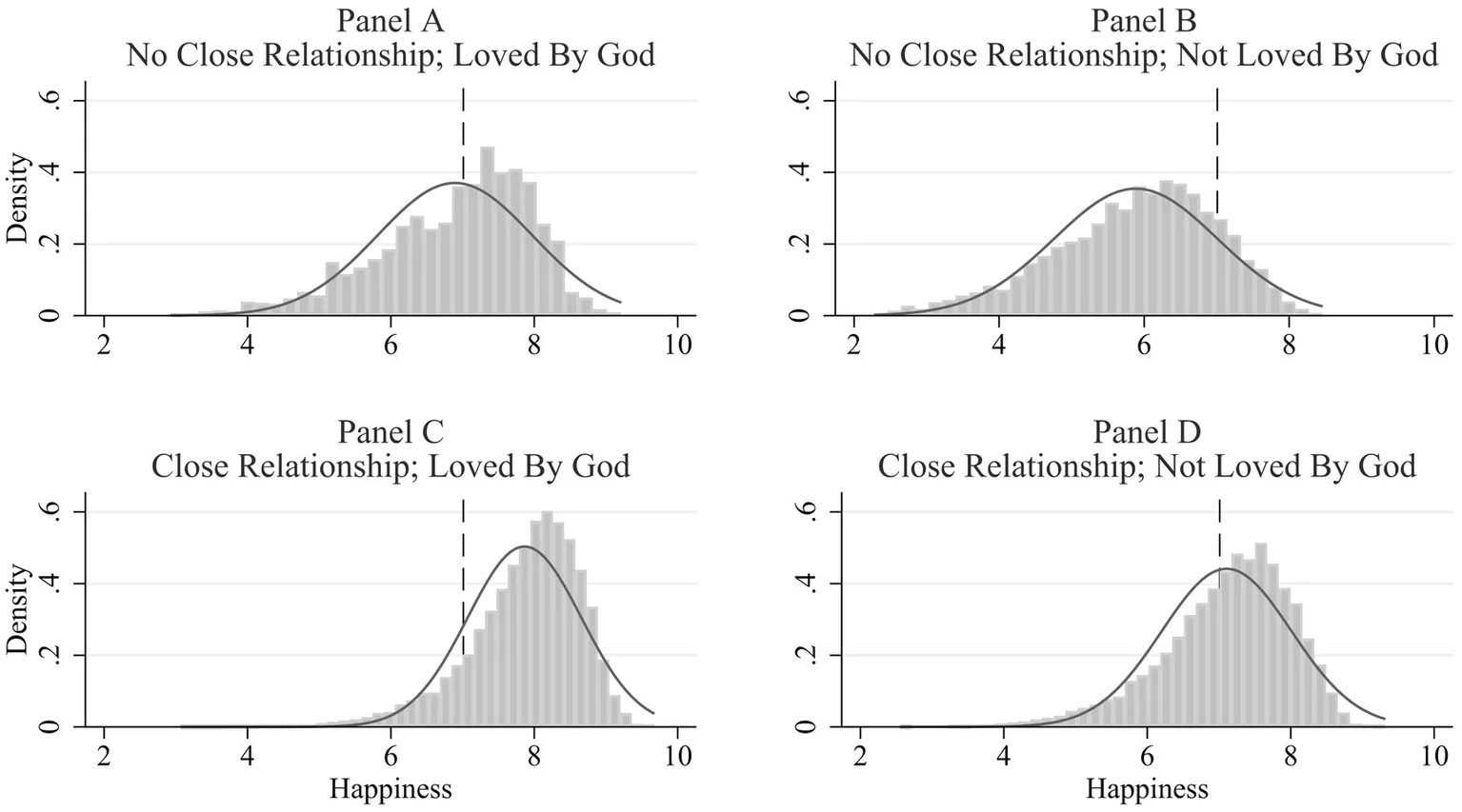
Predicted happiness by close relationship and love by God. Dashed line represents overall mean happiness.

While Figure 1 plots predicted mean happiness, Figure 2 plots the predicted distribution of happiness across four groups, based on whether individuals perceived love by God and whether they had a close relationship. The x-axis displays the values of the happiness scale, while the y-axis displays the density of responses. The vertical dashed line marks the weighted mean happiness for the overall sample. Panels A and B depict happiness distributions for individuals who lacked a close social relationship. Panel A includes respondents who perceived Love by God, while Panel B includes those who did not. The differences between these two panels are striking: individuals in Panel A generally reported higher happiness levels than those in Panel B, whose responses were more dispersed and clustered more heavily below the overall mean. Thus, perceptions of Love by God buffer against the negative effects of the absence of close relationships.

In contrast, Panels C and D show the distribution of happiness for individuals who reported a close relationship. Panel C includes those who felt loved by God, while Panel D displays those who did not feel loved by God. These distributions appear more condensed than the distributions for individuals who did not report a close relationship. They both have distributions where the peak is at or above the mean, and the distributions are remarkably similar. This suggests that the presence of a close relationship mitigated differences in happiness regardless of perceptions of love by God.

Yet, there is also a clear cumulative effect. Panel C, representing both a close relationship and love by God, presents the most condensed distribution. Notably, very few cases in the Panel C distribution fall below mean levels of happiness. In contrast, the absence of both close relationships and love by God (Panel B) is associated with the greatest dispersion of happiness with a large skew below mean levels of happiness. This demonstrates the compounding negative effects of lacking both social and spiritual relationships.

## 4 Discussion

This study, based on U.S. data from the Global Flourishing Study, found that love by God moderates the well-established relationship between close social relationships and happiness. This research offers a novel contribution, as few studies have explored the role of Godly love in shaping happiness in light of a person’s close relationships. Close relationships are, themselves, a manifestation of love that results from both interconnection and a decision to maintain a relationship with the other. Pitirim Sorokin’s complex and often overlooked theory of love (1964), developed decades ago, argued that love—especially Godly love—can influence social cohesion and individual wellbeing.

Notably, the analysis showed that once a close relationship was present, perceptions of love by God do not further enhance happiness. Individuals with close relationships reported similarly high levels of happiness, regardless of whether they perceived Divine love. However, for those without close relationships, feeling loved by God provided resilience, helping individuals maintain happiness despite the absence of close personal connections. This finding contributes to research on social relationships by illustrating a condition under which the lack of a close relationship may not function as a stressor, as manifested in levels of happiness. Importantly, although perceptions of divine love buffer against the negative effects of lacking a close relationship, the most favorable outcomes—the highest and most condensed distribution of happiness—were observed among individuals who report both a close relationship and divine love. This cumulative pattern suggests that close relationships and perceptions of God’s love operate as complementary, rather than fully interchangeable, sources of resilience.

Given that this analysis was based on secondary data, the measurement of love by God was limited to a single measure. Future research should extend this measure of love to fully capture Sorokin’s conceptualization as it is a two-way relationship, suggesting the need for a measure that captures perceptions of love for and by God. Not only was the original conceptualization two-directional, it was multidimensional (60, 61). Levin (39, 62) developed a multidimensional measure of religious love that should be further incorporated into Sociological research. While the present study does not include the broader multidimensional measure as it relies on secondary data, it is important to note that Levin’s measure of religious love had an alpha of 0.92, indicating an extremely high internal consistency between the components, suggesting that the single item measure presented in this study would likely be highly correlated with other items in Levin’s measure. Future research should incorporate a multidimensional measurement of religious love. In the meantime, this study illustrates that perceiving love by God plays a role in offering resilience to individuals who lack close social relationships.

This study highlights the need for future research to further integrate Sorokin’s conceptualization of religious love into sociological inquiry. Sorokin’s proposition that religious love acts as a buffer against societal challenges in modernized contexts is profound and merits deeper exploration. Given the limited existing research on religious love, there is substantial opportunity for future studies to extend this work. For instance, researchers could examine whether religious love fosters resilience in the face of significant life events such as failure, status loss, divorce, job loss, or incarceration. Future studies could also investigate both social isolation and loneliness as potential moderating factors. Comparing the effects of religious love with other well-studied forms of love, such as parental or romantic love, may offer additional insights into the diverse sources of resilience. Moreover, there is potential to expand this analysis by exploring variations in the experience of divine love across different religious traditions and cultural contexts. Refining the conceptualization of close relationships—by considering their length, type, and depth—would also strengthen this line of inquiry. Once broader measures of love and relational dynamics are incorporated, researchers could assess the extent to which different forms of love may serve as substitutes for one another in promoting happiness. Finally, expanding the outcome measures to include additional dimensions of flourishing, such as mental and physical health, would further advance understanding of the broader impacts of religious love.

## Data Availability

Publicly available datasets were analyzed in this study. This data can be found at: https://www.cos.io/gfs-faqs.
